# Electrophysiological evidence of preserved hearing at the end of life

**DOI:** 10.1038/s41598-020-67234-9

**Published:** 2020-06-25

**Authors:** Elizabeth G. Blundon, Romayne E. Gallagher, Lawrence M. Ward

**Affiliations:** 1 Department of Psychology, Vancouver, Canada; 2 Department of Family Medicine, Vancouver, Canada; 3 Djavad Mowafaghian Centre for Brain Health, University of British Columbia, Vancouver, Canada; 4 Department of Family and Community Medicine, Providence Health Care, Vancouver, Canada

**Keywords:** Auditory system, Consciousness

## Abstract

This study attempts to answer the question: “Is hearing the last to go?” We present evidence of hearing among unresponsive actively dying hospice patients. Individual ERP (MMN, P3a, and P3b) responses to deviations in auditory patterns are reported for conscious young, healthy control participants, as well as for hospice patients, both when the latter were conscious, and again when they became unresponsive to their environment. Whereas the MMN (and perhaps too the P3a) is considered an automatic response to auditory irregularities, the P3b is associated with conscious detection of oddball targets. All control participants, and most responsive hospice patients, evidenced a “local” effect (either a MMN, a P3a, or both) and some a “global” effect (P3b) to deviations in tone, or deviations in auditory pattern. Importantly, most unresponsive patients showed evidence of MMN responses to tone changes, and some showed a P3a or P3b response to either tone or pattern changes. Thus, their auditory systems were responding similarly to those of young, healthy controls just hours from end of life. Hearing may indeed be one of the last senses to lose function as humans die.

## Introduction

In the last hours before an expected natural death many people enter a period of unresponsiveness, during which they no longer respond to their external environment. This can be a profound and spiritual time for families, but it is currently unknown whether unresponsive patients are aware of the touch or words of their loved ones. There is a persistent belief, however, that some unresponsive patients may still be aware of touch and sound^1^, despite being unable to reliably signal their awareness. Much of this belief comes from reports of near-death experiences (NDEs), where a common recurring element of this experience is hearing unusual noises or hearing oneself pronounced dead^2–4^. Reports from NDEs, however, are difficult to interpret, because incidence of NDEs is low, between 6%^5^ and 12%^6^ of cardiac arrest survivors, and the cognitive neuroscience underlying NDEs remains hotly debated^7–9^ and poorly understood^10–12^. Further perpetuating the belief that “hearing is the last to go” are some family members and health care providers who have reported that unresponsive patients will occasionally groan or make a small facial movement in response to hearing a familiar voice, but to our knowledge there is no empirical evidence to corroborate these anecdotes^13^.

## Is it possible for a dying brain to sustain awareness?

Neuroprotective mechanisms, mainly the blood-brain barrier, reduce neuronal firing in response to ischemia (a common physiological cause of unresponsiveness at the end of life), which could protect the brain from irreversible brain damage under these conditions^14^. The brain’s tolerance to ischemia has been demonstrated in autopsy, as only about 60% of patients who had been declared brain dead before death showed signs of moderate to severe cortical ischemia, and only about 30% in deep brain structures such as the thalamus and basal ganglia, and a similar percentage in the cerebellum^15^ (see also the following responses^16,17^). The brain, therefore, may be somewhat resistant to the effects of ischemic damage while the rest of the body shuts down just before death. In addition, opioids can reduce behavioural responses to external stimulation, without necessarily reducing awareness^18^. Pain and shortness of breath are common symptoms among the physiological changes that occur at the end of life^19–21^, and are frequently controlled with opioids^22–24^. Patients who are being treated for pain with opioid medications could, therefore, become less responsive to their external environment as they enter the final stage of dying, but may retain some covert awareness. Finally, a surge of cortical gamma power and connectivity is present in the rat brain for 30 seconds immediately following cardiac arrest^25^ (see also the following response^26^). Because synchronous gamma oscillations have been linked to conscious cognitive processing in humans^27–31^, increased gamma synchrony could generate an NDE immediately after cardiac arrest (this interpretation is, however, debated)^26^. Although these studies point to the potential for awareness in the dying brain, they speak to neurophysiological states after sudden cardiac arrest, and may not be generalizable to the period of unresponsiveness that can occur before death from other “natural” causes. In a scoping review of 39 human and animal studies investigating brain activity in the period before cardiac arrest, Pana *et al*.^32^ found that “[t]here are no studies describing clinical brain function in the context of progressive hypoxia or ischemia leading to circulatory arrest (pg 79)”. Whereas there is some, but limited, physiological and anecdotal evidence to support the assertion that “hearing is the last to go”, the capacity for awareness during the unresponsive period leading to a “natural” death remains unknown.

## How can we assess awareness in the dying brain?

The standard neurologist’s method of measuring consciousness is to evaluate brain function indirectly by observing behavioural responses to physical stimulation and verbal command^33,34^. Example assessment behaviours include eye opening (arousal/vigilance), visual pursuit (visual functioning), and response to verbal request (auditory and language functioning)^33,35^. Behavioural signs of consciousness can, however, be difficult to detect, particularly among TBI patients, because such behaviours are often “impaired, inconsistent, or easily fatigued”^33^ [see also^36^]. There has been growing concern, therefore, that assessment of consciousness (and subsequent prognosis) using only behavioural measures is disturbingly inaccurate^37–44^, with misdiagnosis rates estimated at up to 43%^45–48^. Recently, some patients with disorders of consciousness (DOC) (who were diagnosed using conventional behavioural measures) have shown signs of residual cognitive function by means of neuroimaging^49–55^. Neuroimaging methods of consciousness assessment more directly evaluate brain function by observing neural responses to verbal command^40,44,56–61^. For example, in a seminal fMRI study, one DOC patient, who had not previously demonstrated any behavioural signs of awareness of her environment, showed patterns of BOLD activation consistent with controls when she was asked to engage in motor and visual-spatial mental imagery^55^. This was the first demonstration of possible covert awareness among behaviourally unresponsive TBI patients^62^. Subsequent studies have uncovered neural signs of covert awareness among DOC patients using fMRI^52,63–65^, EEG^49,66–68^, and PET^69–71^. Neuroimaging is a useful tool to assess awareness among behaviourally unresponsive TBI patients^54,72–75^, the application of which could be extended to unresponsive patients at the end of life.

**Figure 1:**
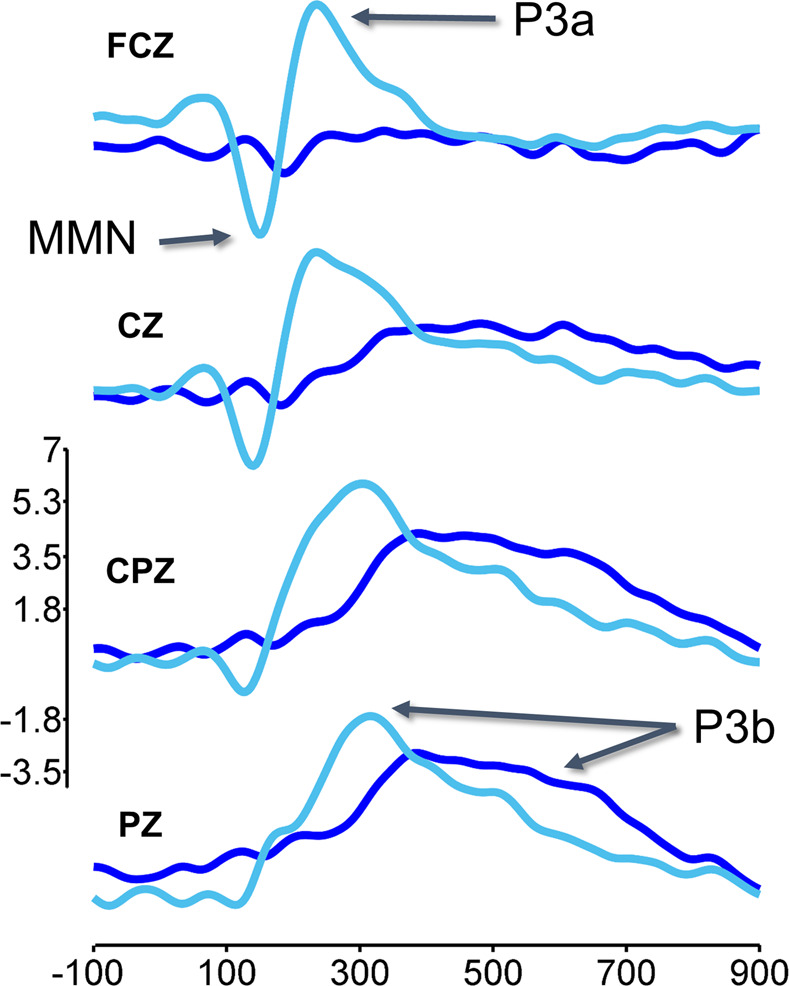
Midline ERPs to tone changes (light blue) and pattern changes (dark blue) runs. P3a is typically maximal over central electrodes (FCZ and CZ), and peaks approximately 200–300 ms post stimulus. The P3b is typically maximal over parietal electrodes (CPZ and PZ), and peaks approximately 300 ms post stimulus;^80^ ERP data from ref. ^81^.

The present study consists of a conceptual replication of a study that has demonstrated evidence of command following in individual healthy control participants and minimally conscious participants, but not unresponsive wakeful patients^49^. The results of the original study revealed that both minimally conscious and (most) unresponsive wakeful patients generated an MMN or P3a (which they called a “local effect”) to simple tone changes, but only minimally conscious participants generated a P3b (which they called a “global effect”) to changes in auditory patterns. The MMN and P3a have been widely implicated with pre-attentive and pre-conscious processing of acoustic irregularities^76–78^, although the MMN does seem to operate on a consciously accessible memory trace^79^. The P3b, on the other hand, seems to always be associated with conscious awareness of task-relevant oddball targets^49,80^. In addition, we have previously shown that this paradigm generates a reliable P3a to tone changes, and P3b to both tone and pattern changes, among control participants^81^ (see Fig. 1 for control MMN, P3a and P3b ERPs). The P3a and P3b are subcomponents of the P300 response^80,82^ and are associated with different attentional processes. The P3a is associated with exogenous attention orienting toward highly salient, rare, and unpredictable oddballs, whereas the P3b is associated with context updating and working memory processes relevant to target detection^83^. Whereas the P3a and MMN often co-occur, whether the P3a operates on unconscious or conscious memory traces is also currently a matter of debate^84–86^. The P3b, however, is considered a reasonably reliable marker of conscious detection of target stimuli^49,80,87,88^, though this interpretation has recently been debated^89–91^.

By applying a similar paradigm to actively dying unresponsive patients we can (tentatively) infer the capacity for unresponsive patients to engage in auditory perceptual processes (MMN), as well as exogenous (P3a) and endogenous (P3b) attentional processes. “Actively dying” is defined as the process taking place during “the hours to days preceding imminent death during which time the patient’s physiologic functions wane.”^92^ In the present study we report the MMN, P3a, and P3b responses to auditory tone and pattern changes both from young, healthy control participants, from responsive hospice patients, and from unresponsive, actively dying, hospice patients. To properly appreciate the results of this study, however, it is important to understand that even the responsive hospice patients differed from the controls in several respects. First, they were all terminally ill (usually cancer; see Supplementary Tables S2a, S2b) and were being medicated (Supplementary Tables S2a, S2b), often with opioids, to ameliorate the symptoms of the illness. Second, they were as a group considerably older than the controls, on average by nearly five decades. All these factors can be expected to affect patients’ EEG responses in the auditory task.

**Figure 2:**
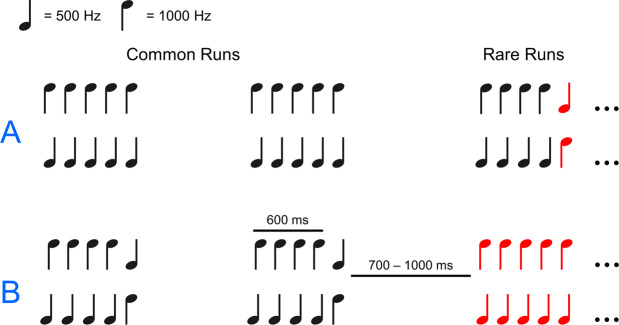
Stimuli and study design. Stem-down notes are 1000 Hz and stem-up notes are 500 Hz. Flat runs consisted of five pure tones of the same frequency; change runs consisted of four pure tones of the same frequency followed by a fifth tone of a different frequency. Participants each heard four sequences. In two of the sequences participants were instructed to search for rare change runs among common flat runs (**A**), whereas in the other two sequences participants were instructed to search for rare flat runs among common change runs (**B**). Rare runs are targets to be detected in the longer sequence of common runs.

All participants listened to oddball sequences, where stimuli consisted of two different types of five-tone auditory patterns: “change runs”, which contained a frequency deviant, and “flat runs”, which did not (see Materials and Methods; see also Fig. 2). In half the oddball sequences flat runs were common and change runs were rare targets, and in the other half change runs were common and flat runs were rare targets. Control participants pressed a button when they heard a rare run, patients silently counted the rare runs (as they would not be able to press a button when unresponsive). MMNs were defined as early (0–300 ms) fronto-central negativities to any frequency deviant (i.e. all change runs compared to all flat runs, regardless of whether the runs were common or rare). P300s were defined as late (200–700 ms) fronto-central (P3a) or centro-parietal (P3b) positivities to rare target patterns compared to common patterns. The “tone change” condition compared change runs when they were common to when they were rare, and the “pattern change” condition compared flat runs when they were common to when they were rare. Hit rates and reaction times to rare patterns could only be recorded for control participants (described extensively in Ref. ^81^.).

## Results

**Figure 3:**
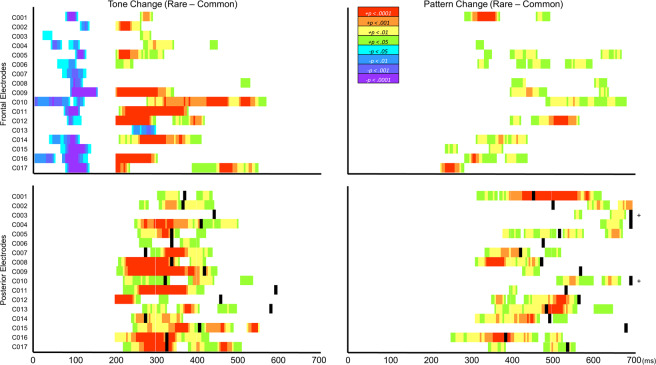
Individual differences in ERP and reaction time (RT) responses from control participants (C001-C017) to local tone deviants (left) and global pattern deviants (right). Warm colours (green through red) represent positive deflections in the difference wave for tone and pattern deviants when they were rare targets (rare – common). Cool colours (blue through purple) represent negative deflections in the difference wave for all tone deviants regardless of whether they were common or rare (change – flat). Black represents each participants’ approximate reaction time. Black time points with a plus sign (+) outside the grid represent RTs that were longer than the last time point in the grid. Each row represents a participant (participant ID is listed to the left of each row). MMN (cool colours) and P3a (warm colours) responses were measured from the fronto-central electrode, and P3b (warm colours) responses were measured from the centro-parietal electrode, where the response to the rare run (or change run for MMN) was largest for each participant (see Supplementary Table S1 for the list of electrodes used in this analysis). Only meaningful time point clusters (p < 0.0003 determined from permutations cluster test) within the MMN (0–300 ms) and P300 (200–700 ms) timerange are shown. Colours correspond to the probability (p value) that the difference between rare and common runs (or change and flat runs) at that time point is 0.

**Figure 4:**
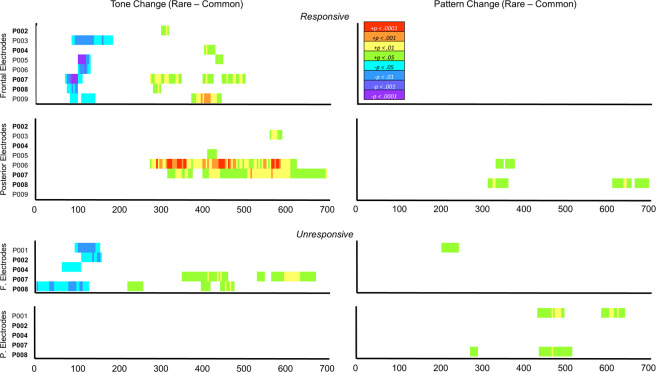
Individual differences in ERP responses from both responsive (top panel) and unresponsive hospice participants (P001-P009) to local tone deviants (left) and global pattern deviants (right). Warm colours (green through red) represent positive deflections in the difference wave for tone and pattern deviants when they were rare targets (rare – common). Cool colours (blue through purple) represent negative deflections in the difference wave for all tone deviants regardless of whether they were common or rare (change – flat). Each row represents a participant (participant ID is listed to the left of each row). MMN (cool colours) and P3a (warm colours) responses were measured from the fronto-central electrode, and P3b (warm colours) responses were measured from the centro-parietal electrode, where the response to the rare run was largest for each participant (see Supplementary Table S1 for the list of electrodes used in this analysis). Only meaningful time point clusters (p < 0.0003 determined from permutations cluster test) within the MMN (0–300 ms) and P300 (200–700 ms) time range are shown. Colours correspond to the probability (p value) that the difference between rare and common runs (or change and flat runs) at that time point is 0.

A participant was deemed to have generated a local or global effect if there were at least 5 consecutive timepoints (cluster) where the conditions were significantly different (*p* < 0.05, 1-tailed). Cluster significance was further evaluated by means of a cluster permutations test. Only meaningful clusters at *p* < 0.0003 (Bonferroni correction) are shown in Fig. 3 & 4. The main results are summarized in Table 1.

**Table 1:** Summary of the proportions of participants that show evidence of each ERP.

	MMN	Tone Change		Pattern Change	
		P3a	P3b	P3a	P3b
Control	1	0.82	0.94	0.76	0.88
Responsive	0.75	0.75	0.50	0	0.25
Unresponsive	0.80	0.40	0	0.20	0.40

### Controls

All (17/17; 100%) control participants showed some evidence of early (0–300 ms) frontal negativity (MMN) or late (200–700 ms) fronto-central positivity (P3a) to tone changes (see Fig. 3 for an overview of individual ERP and RT results for the control group). Similarly, most generated a late centro-parietal positivity (P3b) to tone changes (16/17; 94%) or to pattern changes (15/17; 88%). Several (13/17; 76%) control participants generated a late fronto-central positivity to pattern changes, but many of these responses were weak (0.5 < *p* < = 0.1), and, like the centro-parietal responses to pattern changes, the latencies of the responses varied considerably more than those to tone changes. Hit rates were at or near ceiling for most participants (93–100%), save for participant C011, who only detected 29% of the tone changes, and participants C003 and C015, who detected 78% and 59% of pattern changes, respectively. Reaction times (RT) and P3b peak latencies appear to co-vary (Fig. 3; statistics not reported due to insufficient variability in the RT and peak latency data).

### Responsive hospice patients

Like control participants, all responsive hospice patients showed some evidence of either an early frontal negativity (MMN), or a late fronto-central positivity (P3a), or both, to tone changes. By contrast, only half (4/8; 50%) showed some evidence of late centro-parietal positivity (P3b) to tone changes. Latencies of both fronto-central and centro-parietal positivities to local tone changes were, in general, longer among responsive hospice patients compared to controls. Very few (2/8, 25%) responsive patients showed evidence of centro-parietal positivity (P3b), and none showed evidence of fronto-central positivity, to pattern changes.

### Unresponsive, actively dying, hospice patients

Consistent with control and responsive participants, all (5/5) showed some evidence of either an early fronto-central negativity (MMN), or a later fronto-central positivity (P3a), or both, to tone changes. None of the unresponsive patients showed a centro-parietal positivity (P3b) to the tone changes. One unresponsive patient (20%) showed evidence of a weak late fronto-central positivity (P3a) to pattern changes, and two patients showed stronger centro-parietal positivity (P3b) to pattern changes. See Fig. 4 for an overview of the results for both responsive and unresponsive hospice patients.

**Figure 5:**
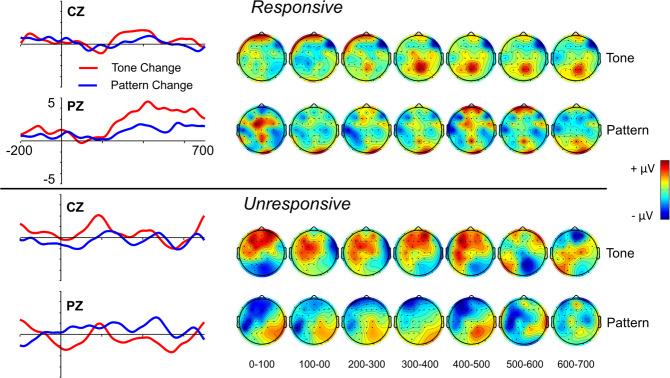
Patient group ERP difference waves and Scalp Maps for responsive (top, n = 8) and unresponsive (bottom, n = 5) patients. Topographic data were averaged across each 100 ms time interval. ERPs were filtered at 10 Hz.

Responsive and unresponsive patient ERP results are also reflected in the group average responses (see Fig. 5). Group average P3a response was weak, but observable, to tone changes, but not to pattern changes, for both responsive and unresponsive patients. P3b response to tone changes was much stronger than to pattern changes among responsive patients, and no P3b response was observed to either tone or pattern changes among unresponsive patients. See also Figures S2-S4 for individual patient scalp maps, and Figures S5-S9 for ERPs and scalp maps of the four patients who contributed data to both responsive and unresponsive sessions.

It should be noted here that we are presenting scalp maps and ERPs of individual patients in Figures S1-S8. “Typical” ERP morphology and latency are based on averaged data from many trials from many participants (“grand average ERPs”). Individual ERPs, even averaged over many trials, seldom replicate the grand average ERPs. Thus, the individual scalp maps and ERPs need to be interpreted with this in mind. In particular, when comparing the ERPs of patients whom we recorded both when responsive and unresponsive (Figures S4-S8), it will be seen that although some display typical morphology, others do not, although they do display difference waves that indicate different responses to the different run types, as indicated.

**Figure 6:**
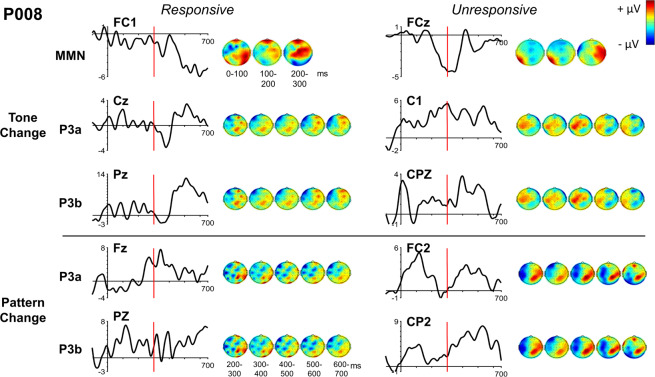
Individual ERPs and Scalp Maps for P008. Topographic data were averaged across each 100 ms time interval. Scalp maps are scaled relative to their own minimum and maximum values. Only ERP difference waves from – 800 ms to 700 ms from the last tone of the run are shown. Difference waves were baseline corrected from −800ms to −600ms from the last tone of the run (i.e. −200 to 0 ms from the first tone of the run) and filtered at 10 Hz. Black bars represent the onset of the first tone of the run (−600ms), red bars represent the onset of the last tone of the run (0 ms).

One example of particular interest, in which the ERP morphology of a patient when unresponsive is inconsistent with typical characteristics, is the fronto-central negativity to tone changes (MMN) measured from P008 (see Fig. 6). This negativity may not be in response to the final deviant tone of the run, as scalp potential begins to decrease before the onset of the last tone of the run, yet it persists for 200 ms after the deviant tone. This morphology cannot be attributed to an idiosyncrasy of P008’s neural activity, as the morphology of their fronto-central negativity to tone changes was more consistent with conventional MMN activity when they were responsive. A similar observation can be made regarding P008’s fronto-central positivity to tone changes, as their fronto-central positivity was more consistent with typical P300-like activity when they were responsive than when they were unresponsive (Fig. 6). Surprisingly, P008’s centro-parietal positivity to pattern changes is more consistent with typical P300-like activity when they were unresponsive than when they were responsive (Fig. 6). Because the difference waves to tone changes measured from P008 don’t resemble a typical MMN or P3a, the significant time point differences reported in Fig. 4 may not indicate that P008 was aware of those tone changes. Differences in ERP activity between rare and common runs (or change and flat runs) do, however, indicate cortical differentiation between the conditions.

## Discussion

We have presented evidence that at least a few actively dying hospice patients, when they are unable to respond to family or healthcare provider verbal stimuli, nonetheless seem to be hearing and giving neural responses to sequences of simple auditory stimuli. This is consistent with the trope that hearing is one of the last senses to lose function when a person is dying, and lends some credence to the advice that loved ones should keep talking to a dying relative as long as possible. Importantly, we were able to discern a “local effect,” either an MMN or a P3a or both, to tone changes in 100% (17/17) of controls, and a “global effect,” namely a P3b, to both tone and pattern changes in most (88–94%) controls. For comparison, using their highly similar paradigm, Bekinschtein *et al*.^49^ were able to detect both local and global effects in all 11 of their control participants who counted oddballs. It should be noted, however, that although Bekinschtein *et al*.^49^ defined local and global effects similarly, they did not clearly differentiate between P3a and P3b effects by separating analyses of stimuli expected to produce the two effects (here tone change and pattern change). They simply combined all rare global targets, whether tone change or pattern change, together in their analyses of the global effect, similarly to how they (and we) combined all local tone change stimuli together in analyses of the MMN. Moreover, they assessed responses recorded at several (10 or 20) electrodes to measure both local and global effects. In contrast, we analysed responses to tone change and pattern change stimuli separately and observed the effects at specific electrodes where P3a and P3b effects are typically largest. This latter approach allows a more precise inference about which underlying brain processes are likely still functioning effectively. Nonetheless, in combination with the results of Bekinschtein *et al*.^49^, it seems that this paradigm is a valid one for indexing functioning of auditory change detection networks in young, healthy participants, which implies that similar results with unresponsive patients can be taken to indicate functioning of these networks.

Our results for responsive and unresponsive patients are also consistent with reports that minimally conscious state (MCS) and unresponsive wakefulness state (UWS, formerly vegetative state, or VS) patients in a similar auditory search paradigm evidence local effects, and a few even show global effects^49^. Because control participants who were engaged in mind wandering or a distracting task seldom showed global effects, this implies especially that when global effects are seen in an individual participant, in particular the P3b, there is some reason to believe conscious awareness of the stimuli is mediating responses. At the least, the presence of local and/or global effects in a DOC or unresponsive patient in this paradigm implies that auditory change networks in these patients are functioning similarly to those of young, healthy participants. Moreover, a few patients in UWS also give fMRI evidence that they can control their brain responses on request^44,49–54,62^. Thus, either when severely damaged or even when near death, some brains can evidence functioning in some systems.

It is worth pointing out that even some young, healthy, participants failed to generate clear P3b responses to auditory change targets, and that the absence of these P3b responses does not imply that the participant was not attending to the targets. Recall that four control participants failed to generate detectable P3b responses to either tone or pattern changes, but neither reduced accuracy nor longer reaction time consistently co-occurred with the missing P3b responses (see Fig. 3 and results section). In fact, for 3 of these participants, hit rates in the condition with the missing P3b were near ceiling (94–100%). This suggests that should a participant fail to generate a P3b response to auditory change targets during a single EEG recording session, this does not imply that they were not able to detect the targets during that session. Furthermore, one participant (C011) generated a clear P3b response to tone changes despite having only behaviourally responded to 30% of the targets. This may be due to a lapse in focused attention (somnolence?) in the middle of each tone change block, as their accuracy was high at the beginning and the end of those blocks, but they stopped responding in the middle.

These inconsistencies in performance among young, healthy participants can inform our interpretation of the hospice patient performance. For example, both P001 and P008 generated a P3b to pattern changes, but not to tone changes, when they were unresponsive. This implies a rather shocking result; that these participants were able to perform the more difficult task of counting rare patterns that contained no salient feature but could not perform the much easier task of counting rare patterns that contained a highly salient feature. While this pattern of responses was atypical among responding controls, C003 showed a similar pattern to P001, where they failed to generate a P3b to tone changes but generated a P3b to pattern changes. Furthermore, C003’s behavioural accuracy in the tone change condition was much higher (100%) than in the pattern change condition (78%). Moreover, P008 also showed this pattern of ERP activity (P3b to pattern but not to tone changes) when they were responsive. This means that the pattern of responding demonstrated by both P001 and P008 when they were unresponsive is consistent with a pattern of responding demonstrated by a conscious, healthy brain, and a conscious hospice patient. It is, therefore, plausible, that both P001 and P008 were aware of their auditory environment at the end of their life.

It is still unclear how the partial functioning of the auditory change detection system we have reported here relates to normal conscious awareness, in spite of arguments from the existing literature^52,93^. We simply do not know how much cortical and sub-cortical functioning is required to support even simple phenomenal conscious awareness. We do know that conscious awareness is not lost even when up to half of cortex is removed during resections done to ameliorate epilepsy, although of course there are clear cognitive deficits in these cases^94^. We also know that in patients with DOC a “cognitive-motor dissociation” can occur^95^. In this state, patients can be behaviourally unresponsive to verbal commands but can display EEG evidence of function in relevant areas of the brain, implicating cognitive function, in response to the same verbal commands. In some cases specific connections between cortical and sub-cortical motor areas have been lost^96^, whereas in others there might be multiple reasons for the lack of motor responsiveness^97^. In the dying brain, it is likely that different areas and connections lose function at different times, with motor loss often preceding cognitive loss, as we seem to have discovered, but there may be no one route to complete loss of function. Clearly though, it is possible that even partial functioning of a cortical-sub-cortical system could result in some awareness even if that awareness cannot be communicated to observers via the usual motor responses.

## Materials and Methods

Some of the following descriptions of the materials and methods, including ethics statement, stimuli, procedure, EEG preprocessing, Independent Component Analysis, and artefact rejection, are very similar to those detailed in ref. ^81,83^. Some analyses include aspects unique to the present experiment. All aspects of the experimental procedure and EEG analysis apply to both responsive and unresponsive sessions of hospice patients and to the single sessions of control participants, unless otherwise specified. All analyses of individual differences in ERP responses are unique to this study. Only details of the participants, materials, procedure, and EEG preprocessing unique to the hospice patients are included. Details of those aspects of the study that are unique to the 17 control participants, all students at the University of British Columbia (ages 18 to 30 years, mean age 21.3 years, 10 females) are described in Ref. ^81^. These details include descriptions of psychophysical (reaction time and latency) and electrophysiological (P3a and P3b latency and amplitude) differences under the different search conditions (see procedure) at the group level. MMN analyses were not included in the previous report.

### Ethics statement

All aspects of the experimental protocol, including the recruitment and consent procedures, were approved by the University of British Columbia Behavioural Research Ethics Board in accordance with the provisions of the World Medical Association Declaration of Helsinki. All participants gave written informed consent by reading and signing the approved consent document. Hospice patients explicitly extended their consent to the time when they became unresponsive. In addition, patients’ families (when available) gave verbal assent to the procedures.

### Hospice patients

Data were collected initially from 13 participants, residing in a hospice facility in Vancouver, over a time period of more than two years. A hospice in British Columbia is a facility that accepts patients with advanced disease with an estimated prognosis of 3 months or less, although prognosis is notoriously unreliable.

Eligibility Criteria: Patients were eligible to participate if they showed no signs of psychiatric or neurological deficits, or cognitive impairment, including delirium, which can be common in the last days to hours. These clinical assessments for eligibility were performed by hospice physicians. In addition, eligible patients must have been proficient in the English language to understand the task instructions, and their hearing must have been sufficient to distinguish between the tone patterns. If there was concern about a patient’s hearing, or if the patient wore a hearing aid, a hearing screening was performed.

We intended to record each patient twice, once as soon as possible after admission to the care facility and given consent, and once when unresponsive and actively dying. Recall that actively dying is defined as “the hours to days preceding imminent death during which time the patient’s physiologic functions wane.”^92^ Time between sessions varied from 8 to 12 weeks, depending on the time elapsed between enrolment in the study (first session) and when the patient became unresponsive prior to death (second session). Typically, an “unresponsive” actively dying patient was defined using the Palliative Performance Scale (PPS) rating of 10%. The PPS is a valid and reliable eleven-point scale designed to measure in 10% decrements the decline from 100% (healthy) to 0% (death) based on five observable parameters: ambulation, ability to do activities, ability to do self-care, food/fluid intake, and consciousness level (Palliative Performance Scale (PPSv2). copyright Victoria Hospice Society, 2006. Accessed at: https://victoriahospice.org/how-we-can-help/clinical-tools/ on August 27, 2019). With respect specifically to consciousness level, the PPS states that ‘full’ consciousness (70% and higher) implies full alertness and orientation with good cognitive abilities in various domains of thinking, memory, etc. Levels 50–60% indicate ‘full or confusion,’ where ‘confusion,’ rated from mild to severe, denotes the presence of delirium or dementia and implies a reduced level of consciousness. Levels 20% to 40% add ‘drowsiness’ to full or confusion, where ‘drowsiness’ indicates either fatigue, drug side effects, delirium or closeness to death. Level 10% indicates drowsiness or ‘coma’, where ‘coma’ means the absence of response to verbal or physical stimuli; with the possibility that some reflexes may remain. The PPS assessment of patients was performed by the nursing staff at the facility. Patient participants were at 70% or higher when we recorded them for the first time. Nurses informed us when a patient had reached the 10% assessment, and we recorded them as soon as possible after this alert.

Between responsive and unresponsive sessions, patients were frequently (approximately every 1–2 weeks) visited by a team member to collect verbal assent to continue to participate in the study. Various forms of attrition, including rapid progression to death or remission, reduced the number of patient participants. One family revoked assent at the time of the unresponsive recording. Data from four patients were excluded because of excessive noise in their EEG. The patient analysis to be described is based on nine patients (four female, age 28 to 88 years, mean age 68.2 years). Eight patients were recorded when they were responsive, five when they were unresponsive. Four patients were recorded once when they were responsive, and again when they were unresponsive, as originally intended. Details of patients’ diagnoses and medications can be found in Supplementary Tables S2a and S2b. Note that most patients were receiving various forms of opioids at both recording sessions. See supplementary Table S3 for information about these medications and their typical usage.

### Stimuli

Our stimuli were identical to those described in^81^; the present description is therefore nearly identical to that in^81^. We generated 50-ms duration pure tones with 7.5 ms onset and offset ramps, using the ascending first half of a Hann window for the onset and the descending second half of the Hann for the offset, using a custom MATLAB (MathWorks, Natick USA) script. The tones were administered binaurally at 70 dB, through insert earphones (EAR 3 A) in a sound-attenuating chamber to controls, and through over-ear headphones to patients in their beds. Stimuli were presented and responses registered using Presentation software (Neurobehavioral Systems Berkeley CA USA). Tone runs were generated using Audacity (Sourceforge). Auditory stimuli consisted of two types of five-tone runs called flat runs and change runs (see Fig. 2). Flat runs consisted of five pure tones of the same frequency while change runs consisted of four pure tones of the same frequency followed by a fifth tone of a different frequency. All runs contained a combination of 500-Hz and 1000-Hz tones which generated two versions of each type of run: one in which 1000-Hz tones comprised the first four tones in a change run and all the tones in the flat run (change-down and flat-high; see Fig. 2B), and the same for the 500-Hz tones (change-up and flat-low see; Fig. 2A). Successive 50-ms duration tones in a run were separated by 100 ms of silence. Each run lasted 650 ms from the onset of the first tone to the offset of the last tone. Intervals between the offset of the final tone of a given run and the onset of the first tone of the next run varied randomly from 700 ms to 1000 ms.

### Procedure

Hospice patient data were collected using a portable EEG system in each patient’s room. Each patient lay in their bed for data collection. The bed was adjusted so that the patient could lie upright comfortably, with their head supported by a rolled towel wrapped around their neck to keep the back of their head clear of the bed. Once the EEG was set up and the patient was fitted with the cap, pictures were taken of the patient from many angles using a digital camera without internet access. These pictures served as a record from which to recreate the conditions of the responsive session as faithfully as possible during the unresponsive session. All photos were deleted once the patient had completed, or was no longer enrolled, in the study.

Our protocol consisted of three sections, only one of which forms the basis of this report. The other two were an unguided imagery task with two alternating commands (imagine walking through your house, imagine singing Happy Birthday), separated by musical interludes that required a judgment of dominant instrument. The entire protocol required about 1 hour to complete. Before the protocol began, each patient performed a short loudness screening to make sure they were comfortable with the loudness of the stimuli and instructions. Next, patients were familiarized with the study stimuli and procedure. All task instructions were presented in written format on a laptop, with simultaneous audio recordings of the instructions presented through over-ear headphones. In the part of the study reported here, again identical to the task in^81^ and thus described in a similar fashion here, each participant, control or hospice patient, heard four extended oddball sequences of tone runs (about 35 mins total) in randomized order per participant. Each sequence began with 30 instances of the common run. From then on, the rare run was presented on a random 20% of occasions among 80% common runs. In each sequence rare runs were heard between 18 and 30 times. There were always at least 2 common runs before and after each rare run. The four sequences of runs consisted of the following: (1) common flat-low, rare change-up; (2) common change-up, rare flat-low; (3) common flat-high, rare change-down; (4) common change-down, rare flat-high. Patients were instructed to count the number of pattern oddballs (rare runs) they heard during each block (differently from^81^). Controls were instructed to click a mouse whenever they heard a rare run (from^81^). It was made clear to participants that they were only to count or to respond to runs that represented a change in the global pattern, i.e. a rare run, not every time they heard a tone that differed from the previous tone. For patients, if at any point during the responsive session the patient needed to ask a question about the procedure, or the patient appeared not to understand the procedure, the recording was paused, and the procedure was clarified. During the unresponsive session, the conditions of the responsive session were recreated as faithfully as possible, including head and bed position, laptop placement, and stimulus loudness. Family members were permitted to stay in the patients’ room during the unresponsive session and were encouraged to talk to their loved one between blocks.

### EEG recording and preprocessing

As similarly described in^81^, control participant EEG signals were digitized at 500 Hz (National Instruments Inc., Vaudreuil-Dorion QC Canada) from a 60-channel electrode cap (Electrocap Inc., Eaton OH USA, International 10  -10 placement) referenced to the right mastoid. Before digitization EEG signals were amplified and analog bandpass filtered from 0.1 Hz to 100 Hz (SA Instrumentation, San Diego CA USA). Eye movements were recorded with four periocular electrodes. All electrode impedances were kept below 10 kΩ (input impedance of the amplifier was > 2 gΩ)

EEG signals from patients were digitized at 2048 Hz (BioSemi, ActiveTwo) from a 128-channel electrode cap (only 64 channels were used, BioSemi equiradial placement) referenced to the CMS electrode. Before digitization, EEG signals were amplified and analog bandpass filtered from 0.1 Hz to 100 Hz. Eye movements were recorded with two periocular electrodes, one to the right and one above the right eye.

As in^81^, All EEG data were analyzed using EEGLAB software^98^. Raw data were down-sampled to 256 Hz (250 Hz for control data) and re-referenced to average reference. Line noise was removed online by applying a notch filter between 55 and 65 Hz. Data were visually inspected for large muscle artifacts. Eye-blink and EMG artifacts were removed using Independent Component Analysis.

### Behavioural and ERP analyses

Reaction times and accuracy to target runs were recorded for control participants only. A complete description of how hits were classified, as well as group level analyses of behavioural data, are reported in Ref. ^81^. Reaction times for individual control participants are reported with the ERP results for this study.

ERP analyses were conducted using ERPLAB^99^ software running in EEGLAB. Artifacts were removed using independent component analysis^100^. The continuous EEG record for each individual participant was epoched from −1000 to +2000 ms relative to the onset of the first tone of each run, low-pass filtered at 30 Hz using a FIR filter, and baseline corrected (−200 to 0 msec re onset of first tone in each run). Shortened segments of these epochs were selected for display purposes. EEG trials containing data with amplitudes greater than 100µV, and step-like artifacts consistent with saccadic eye movements, were removed from further analysis.

Consistent with the analyses performed by Bekinschtein *et al*.^49^, MMNs were characterized as the difference between change runs and flat runs (change – flat), regardless of whether they were rare or common, across all four blocks of stimuli. After trials containing artifacts were removed, MMN data (control or hospice patient) comprised 188 to 284 trials of each run type (382 to 576 trials total). P300s, however, were characterized as the difference between rare and common (rare – common) runs, sorted by run type. In other words, we compared rare vs common change runs (now defined as “tone change” comparison) and rare vs common flat runs (now defined as “pattern change” comparisons). This allowed a determination of the effect of the longer-sequence role alone on the P300 subcomponents, independent of the physical makeup of the run. Because each block contained many more common runs than rare runs, only common runs immediately preceding rare runs were retained for further analysis. After trials containing artifacts were removed, P300 ERPs (control or hospice patient) were based on 29 to 55 rare runs of each type (between 60 and 107 rare trials total).

As our study requires reporting ERP responses for individual participants, we adopted definitions of the latency and scalp topography of the MMN, P3a, and P3b that were amenable to capturing individual differences in ERP spatio-temporal characteristics among control and hospice patients. Whereas the MMN typically peaks between 150 ms and 250 ms post stimulus onset^76^, and the P300 can peak anywhere between 250 ms and 500 ms post stimulus onset^80^, latencies of both ERPs are sensitive to stimulus properties and task demands. For example, MMN latencies are often shorter for larger frequency deviations^76^, and P300 responses to targets that deviate in semantic compatibility are often longer than those to targets that deviate in spatial compatibility^80^. We expected, therefore, that MMN peak latency among control participants could be near the beginning of the typical interval. On the other hand, we expected that the peak latency of P300 responses would be different for each condition. In our previous study we found that P3b peak latency was earlier (by about 135 ms) to tone-change targets than to pattern-change targets among controls^81^, and that P3b peak latency to pattern-change targets was quite late (about 500 ms post stimulus onset). Recall that, in this latter condition, participants were asked to identify targets that were missing a tone change among standards that contained that tone change, making this a difficult search task to perform^81^. Finally, we expected that all ERP latencies for hospice patients could also be later, as opioid medications could slow ERP latencies for some patients. While there is currently insufficient data to predict the effects of active opioid ingestion on MMN or P300 responses, some research has shown longer MMN and P300 latencies in opioid-dependent patients^101–103^. On the other hand, some suggest that this relationship is an endophenotypic marker of substance-abuse, rather than an effect of the drugs themselves^104^. To the best of our knowledge, there is currently no published research exploring the relationship between P300 amplitude and latency in patients being treated for pain with opioids. We, therefore, defined the MMN as an early negativity (0 and 300 ms), and the P300 as a late positivity (between 200 and 700 ms) after the onset of the last tone of each run type.

The MMN and P3a are typically maximal over fronto-central midline electrodes (eg FZ or CZ), whereas the P3b is typically maximal over centro-parietal midline electrodes (eg PZ)^76,80^. Because we expected some individual variability at which electrode each ERP would be maximal^76,105^, we extracted ERP data from the fronto-central (MMN and P3a) and centro-parietal (P3b) electrode where that ERP was both maximal within its timerange (0–300 ms for MMN, 200–700 ms for P3a and P3b), and showed characteristic ERP-like morphology by visual inspection, i.e. had both a positive and negative deflection within its time range, where possible (see Supplementary Table S1 for the list of electrodes used in this analysis). For the MMN, a difference wave was calculated for each participant by subtracting all flat runs from all change runs. Data from the fronto-central electrode with the maximal negative deflection of that difference wave during the MMN timeframe was retained for further analysis. A similar procedure was performed for P300s, where difference waves for each run type (change or flat) were calculated by subtracting common runs from rare runs. For each participant, data from the fronto-central and centro-parietal electrodes with the maximal positive deflections for each difference wave during the P300 timeframe were retained for further analysis. Because the frontal component of the MMN is often localized to anterior electrodes^106,107^, electrodes for the MMN were restricted to fronto-central medial electrodes (midline plus one lateral electrode position, i.e. AF1 to AF2) between AFZ and CZ. The P3a, by contrast, is typically localized more centrally^80,105,108,109^, so fronto-central electrodes for the P3a were restricted to medial electrodes between FZ and CZ. Centro-parietal electrodes for P3b analysis were restricted to medial electrodes between CPZ and POZ.

Meaningful individual ERP difference waves were determined using a modified cluster-based permutations test^110^. First, significant timepoints within individual ERP difference waves were determined by comparing conditions (change with flat for MMN, rare with common for P300s) using a paired *t* test for each timepoint. Similar to the analysis adopted by Bekinschtein *et al*.^49^, only ERP clusters of at least 5 consecutive significant time points (a sustained effect of approximately 20 ms) were retained for further analysis (*p* < 0.05 1-tailed). Clusters calculated from ERP difference waves will hereafter be referred to as “real clusters”. Next, “sham clusters” were computed using the same procedure from surrogate permutations of each comparison (*n* = 200). Sham clusters were retained for further analysis if they contained at least as many time points as the real cluster. To perform the cluster-based permutations analysis, each cluster (real or sham) was represented by the sum of all the *t* values within that cluster. Significance of the real clusters was determined by comparing the representative *t* statistic of each real cluster with a distribution of representative *t* statistics from sham clusters. The proportion of sham *t* statistics that exceeded the real *t* statistic served as each real cluster’s *p* value. A cluster was deemed meaningful if its *p* value was smaller than the Bonferroni corrected alpha value (0.05/173 real clusters = 0.0003). Although only meaningful clusters are reported in Figs 3 and 4, each timepoint within those clusters is represented by its own *p* value so that the reader can appreciate the morphology of each individual difference wave. A significant positive deflection (i.e. a significant real cluster where the rare run was larger than the common run) at a fronto-central electrode was classified as a P3a, whereas a positive deflection at a centro-parietal electrode was classified as a P3b. A negative deflection at a fronto-central electrode (i.e., a significant real cluster where the change run was more negative than the flat run) was classified as an MMN. We adopted a somewhat less conservative version of the analysis employed in the original study because, unlike Bekinschtein *et al*.^49^, our goals were not to develop a diagnostically helpful tool for assessing awareness in behaviourally unresponsive patients, but to explore whether some cognitive mechanisms required to support auditory attention may remain functional close to death. In other words, our goals were to see if hearing is even possible for unresponsive, actively dying hospice patients.

## Supplementary information

Supplementary information.

## Data Availability

The datasets generated during and/or analysed during the current study are available from the corresponding author on reasonable request.
