# Supplementary material for: Electrophysiological evidence of preserved hearing at the end of life
Source: Sci Rep. 2020 Jun 25;10:10336. doi: 10.1038/s41598-020-67234-9 (PMC7316981; doi:10.1038/s41598-020-67234-9)
Supplement: Supplementary file 1 — Supplementary information. [file 41598_2020_67234_MOESM1_ESM.pdf]

Electrophysiological evidence of preserved hearing at the end of life

Elizabeth G. Blundon[1], Romayne E. Gallagher[2,4], Lawrence M. Ward[1,3]*

[1]Department of Psychology

[2]Department of Family Medicine

[3]Brain Research Centre

University of British Columbia

[4]Department of Family and Community Medicine, Providence Health Care

	Frontal Electrodes			Posterior Electrodes	
	Change – Flat	Change (Rare – Common)	Flat (Rare – Common)	Change (Rare – Common)	Flat (Rare – Common)
Control					
C001	FCZ	FZ	CZ	P1	PZ
C002	FZ	FCZ	CZ	POZ	CPZ
C003	FC2	CZ	CZ	PZ	PZ
C004	CZ	CZ	CZ	PZ	PZ
C005	FZ	FCZ	CZ	PZ	PZ
C006	FZ	C1	FCZ	PZ	PZ
C007	FCZ	CZ	CZ	POZ	PZ
C008	FZ	FCZ	CZ	P1	P1
C009	FCZ	FCZ	CZ	CPZ	CPZ
C010	CZ	FCZ	CZ	CPZ	CPZ
C011	FZ	CZ	CZ	PZ	PZ
C012	FCZ	FCZ	CZ	CPZ	CPZ
C013	FCZ	FZ	CZ	PZ	P1
C014	FZ	FZ	FCZ	CPZ	CPZ
C015	CZ	CZ	CZ	PZ	PZ
C016	C1	CZ	CZ	POZ	PZ
C017	FCZ	CZ	FCZ	PZ	PZ
Responsive					
P002	AFZ	F2	FCZ	CPZ	PZ
P003	FC1	F2	FZ	PO4	PO4
P004	AF3	C1	FCZ	CP1	CP1
P005	F1	C1	F2	PZ	P1
P006	AFZ	FCZ	CZ	CPZ	PZ
P007	AFZ	F1	CZ	CPZ	PZ
P008	FC1	CZ	FZ	PZ	P2

P009	FCZ	FCZ	C1	CP1	CP1
Unresponsive					
P001	FC1	FCZ	C2	CP1	PZ
P002	F2	FC1	F2	CPZ	PO4
P004	CZ	CZ	F1	CPZ	CP1
P007	F1	FC1	CZ	POZ	P1
P008	FCZ	C1	FC2	CPZ	CP2

Supplementary Table S1: List of electrodes where ERP analyses were performed

Responsive							
Subject ID	Diagnosis	Approx. age at recording	Approx. time of recording	Medication	Dose	Time of Med. Admin.	Method of Med. Admin.
P002	Lung Cancer	66	14:00	Clonazepam<br>Dexamethasone<br>Hydromorphone<br><br><br>Lorazepam	0.25 mg<br>4 mg<br>0.25 mg<br>0.25 mg<br>0.5 mg<br>1 mg	10:00<br>10:00<br>8:00<br>12:00<br>13:25<br>13:25	PO<br>PO<br>SUBCUT<br>SUBCUT<br>SUBCUT<br>PO
P003	Head & Neck Cancer	81	14:00	No relevant medications to report	--	--	--
P004	Lung Cancer	67	13:00	Gabapentin	200 mg<br>200 mg	10:00<br>12:00	PO<br>PO
P005	Astrocytoma	28	14:00	Dexamethasone<br>Levetiracetam	1 mg<br>250 mg	10:00<br>10:00	PO<br>PO
P006	Colorectal Cancer	55	11:00	Dexamethasone<br>Escitalopram<br>Oxycodone	4 mg<br>30 mg<br>90 mg	10:00<br>10:00<br>7:30	PO<br>PO<br>PO
P007	Ovarian Cancer	87	12:00	Escitalopram<br>Fentanyl (patch)	10 mg<br>18 mcg	10:00<br>22:00	PO<br>Transdermal
P008	Lung Cancer	61	12:00	Dexamethasone<br>Hydromorphone	4 mg<br>9 mg	9:00<br>10:00	PO<br>PO
P009	Congestive Heart Failure	77	15:00	Fentanyl (patch)<br>Levothyroxine<br>Methylphenidate	6 mcg<br>25 mcg<br>5 mg	18:00<br>10:00<br>10:00	Transdermal<br>PO<br>PO

Supplementary Table S2a: Details of responsive hospice patient diagnosis and medications consumed on the day of each recording. Locations of cancer metastases are not included. Only medications that may affect cognitive function, and were administered before each recording, are included. Medications not included are those designed to treat the following: moderate pain relief (like acetaminophen and lidocaine), fungal infections, gout, blood clotting, hemorrhoids, constipation, skin diseases, nausea, and heartburn. Two patients (N007 and N009) were

prescribed sleep aids (Zopiclone 15mg PO, and Trazodone 50mg PO, respectively). These medications are not shown as it is unlikely the patients were still affected by the time of the recording based on medication half-lives. Times are listed using 24-hour clock.

List of abbreviations and acronyms: Approx. = Approximate, Med. = Medication, Admin. = Administration, PO = Per Os (medication taken orally), SUBCUT = Subcutaneously (medication administered by injection into the subcutaneous tissue).

Unresponsive								
Subject ID	Diagnosis	Approx. age at recording	Approx. time of recording	Medication	Dose	Time of Med. Admin.	Method of Med. Admin.	Approx. Time between recording and death
P001	Prostate Cancer	88	16:00	Hydromorphone	0.25 -0.5 mg	6:00<br>12:00	SUBCUT<br>SUBCUT	76 hrs
P002	Lung Cancer	66	15:00	Dexamethasone<br>Lorazepam<br>Hydromorphone	4 mg<br>0.5 mg<br>0.5 mg<br>1 mg<br>0.5 mg<br>1 mg	8:00<br>14:00<br>10:00<br>12:00<br>12:45<br>14:30	SUBCUT<br>SUBCUT<br>SUBCUT<br>SUBCUT<br>SUBCUT<br>SUBCUT	6 hrs
P004	Lung Cancer	67	23:00	Dexamethasone<br>Gabapentin<br>Methadone<br>Hydromorphone<br><br><br>Glycopyrrolate	4 mg<br>200 mg<br>7 mg<br>2 mg<br>2 mg<br>2 mg<br>0.2 mg<br>0.2 mg	10:00<br>10:00<br>14:00<br>11:20<br>13:30<br>17:00<br>14:00<br>16:00	SUBCUT<br>PO<br>PO<br>SUBCUT<br>SUBCUT<br>SUBCUT<br>SUBCUT<br>SUBCUT	116 hrs
P007	Ovarian Cancer	87	14:00	Glycopyrrolate	0.2 mg	11:15	SUBCUT	5 hrs
P008	Lung Cancer	61	20:00	Dexamethasone<br>Hydromorphone<br><br><br><br><br><br>Glycopyrrolate	6 mg<br>2 mg<br>2 mg<br>1 mg<br>2 mg<br>2 mg<br>2 mg<br>0.2 mg<br>0.2 mg	10:00<br>10:00<br>14:00<br>14:30<br>17:05<br>18:00<br>20:00<br>14:30<br>20:00	SUBCUT<br>SUBCUT<br>SUBCUT<br>SUBCUT<br>SUBCUT<br>SUBCUT<br>SUBCUT<br>SUBCUT<br>SUBCUT	79 hrs

Supplementary Table S2b: Details of unresponsive hospice patient diagnosis and medications consumed on the day of each recording. Locations of cancer metastases are not included. Only medications that may affect cognitive function, and were administered before each recording, are included. Medications not included are those designed to treat the following: moderate pain relief (like acetaminophen and lidocaine), fungal infections, gout, blood clotting, hemorrhoids, constipation, skin diseases, nausea, and heartburn. For P001 a range of hydromorphone doses is reported because the specific dosage administered was not specified. P004 was able to take medications PO earlier in the day because they did not become unresponsive until the evening on the day of the recording. It was also verbally reported that P004 regained responsiveness sometime after the recording, but no written record of this is available to us. Times are listed using 24-hour clock.

List of abbreviations and acronyms: Approx. = Approximate, Med. = Medication, Admin. = Administration, PO = Per Os (medication taken orally), SUBCUT = Subcutaneously (medication administered by injection into the subcutaneous tissue).

Drug Name	Description and Usage in a Hospice Context
clonazepam	A tranquilizer of the benzodiazepine class usually used for treating persistent anxiety.
dexamethasone	Corticosteroid used in in controlling pain, nausea, raised intracranial pressure and lymphedema.
escitalopram	Antidepressant of the selective serotonin reuptake inhibitor class used to treat major depressive disorder or generalized anxiety disorder.
fentanyl	Opioid used to treat pain or shortness of breath due to advanced disease.
gabapentin	Anticonvulsant medication used to treat neuropathic pain.
glycopyrrolate	Anticholinergic used to reduce salivary production in deeply unconscious patients in order to avoid noisy respiration.
hydromorphone	Opioid used to treat pain or shortness of breath due to advanced disease
levetiracetam	Anticonvulsant medication used to treat seizures secondary to primary tumors of the brain and/or brain metastases.
levothyroxine sodium	Manufactured form of the thyroid hormone thyroxine used to treat thyroid hormone deficiency.
lorazepam	Benzodiazepine medication used to treat anxiety, insomnia, active seizures including status epilepticus,
methadone	Opioid used for pain management, usually when other opioids not tolerated or not effective.
methylphenidate	Stimulant medication used to counter sedation secondary to the use of opioids for pain management or shortness of breath.
oxycodone	Opioid used to treat pain or shortness of breath due to advanced disease.
trazodone	Antidepressant medication used in smaller doses for sleep and anxiety.
zopiclone	Hypnotic agent used in the treatment of insomnia with a similar side effect profile to benzodiazepines.

Supplementary Table S3: List of medications administered to hospice patients summarized in Tables S2a and S2b. List includes a description of each medication and its use in a hospice context

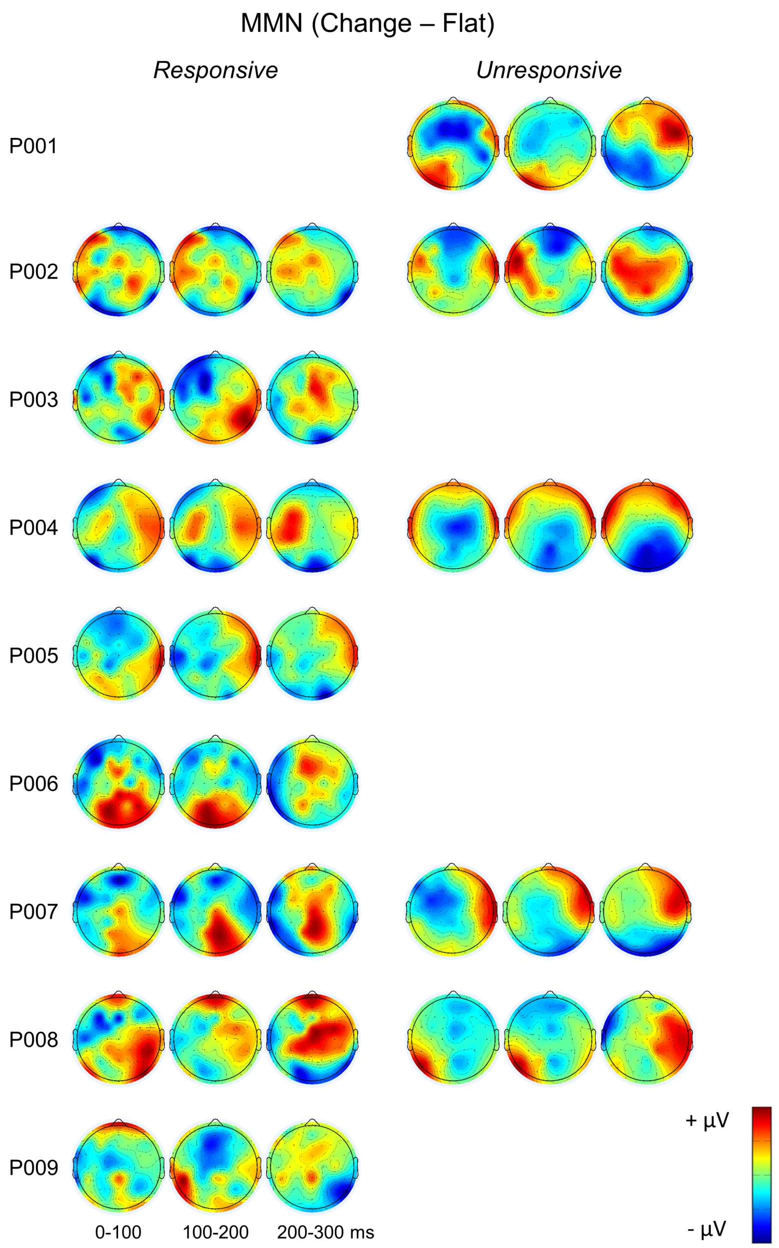

Supplementary Figure S1: Patient Scalp Maps (MMN). Topographic data were averaged across each 100ms time interval. Scalp map are scaled relative to their own min and max values.

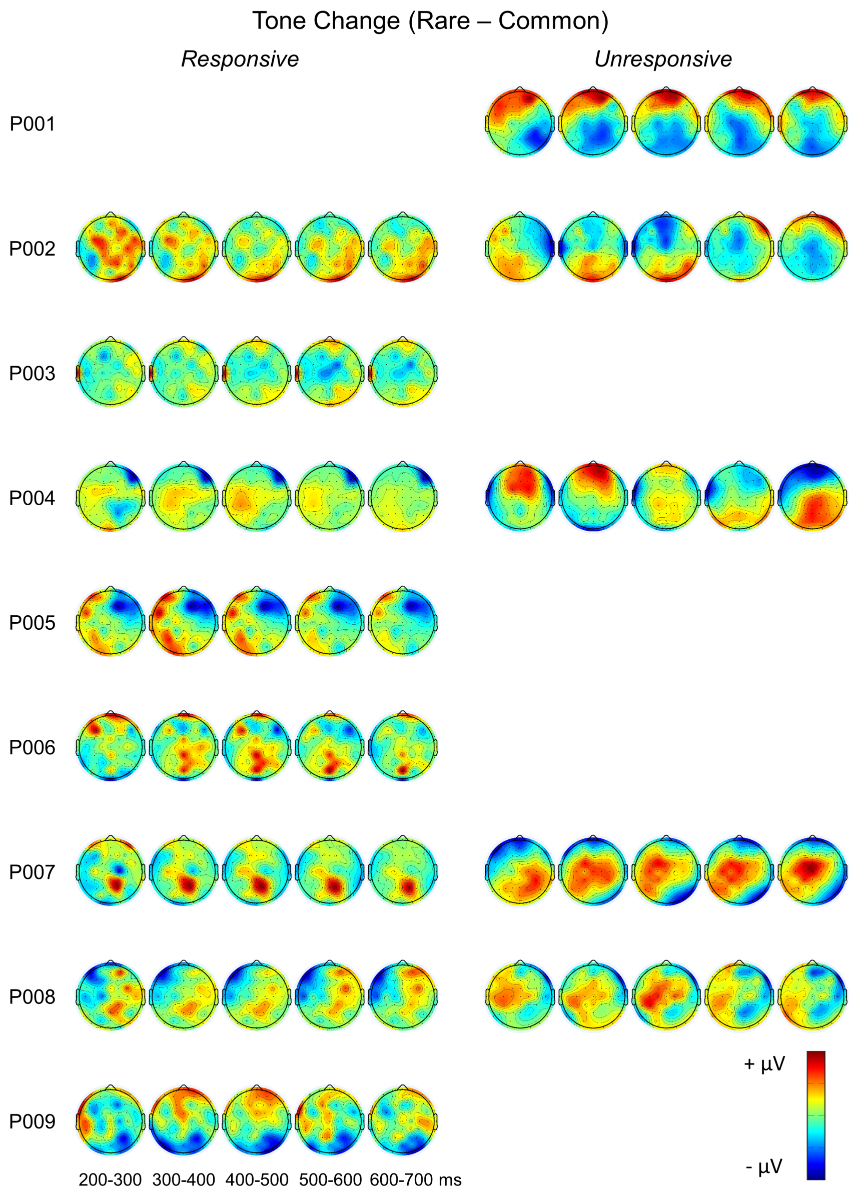

Supplementary Figure S2: Patient Scalp Maps (Tone Change). Topographic data were averaged across each 100ms time interval. Scalp map are scaled relative to their own min and max values.

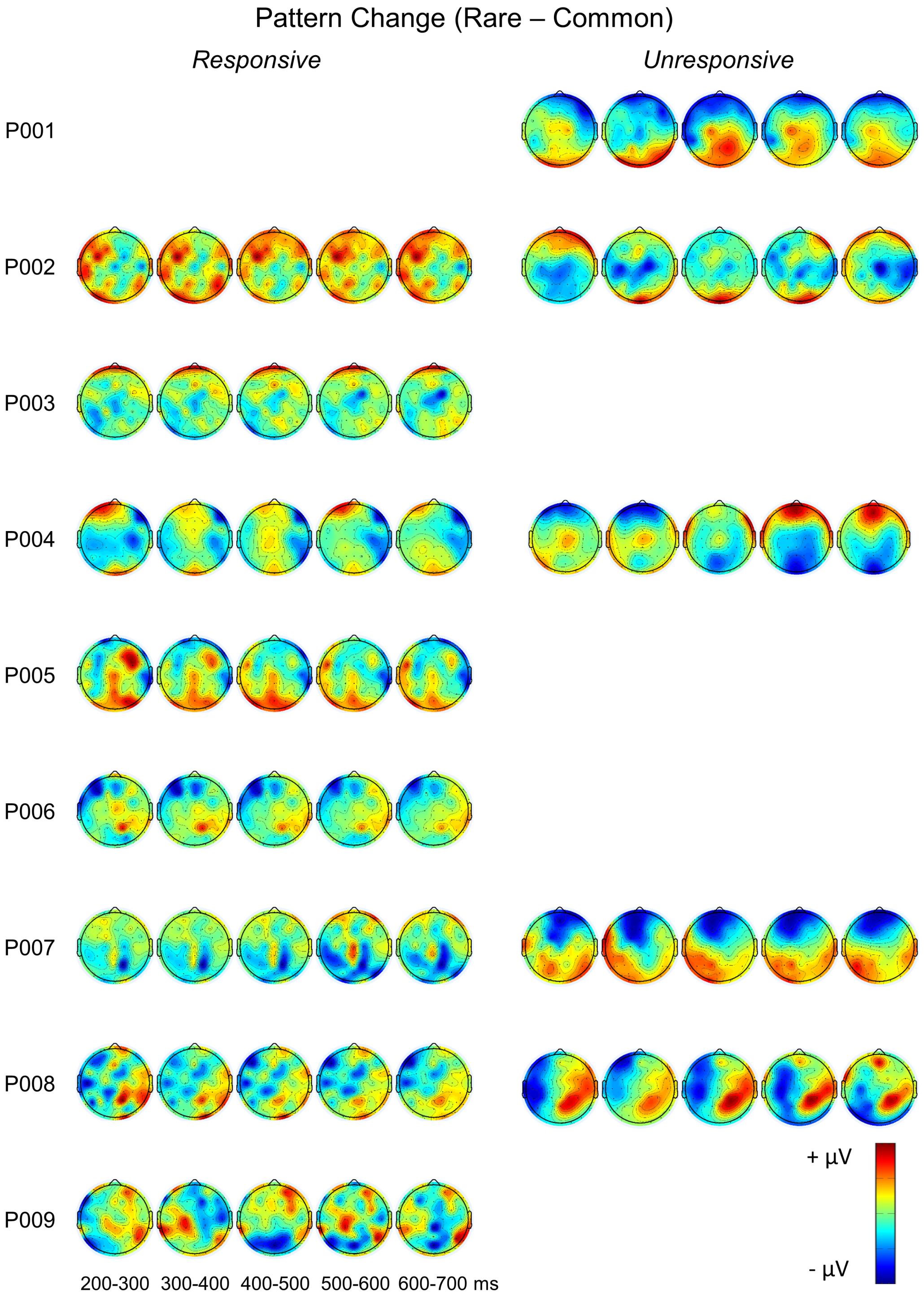

Supplementary Figure S3: Patient Scalp Maps (Pattern Change). Topographic data were averaged across each 100ms time interval. Scalp map are scaled relative to their own min and max values.

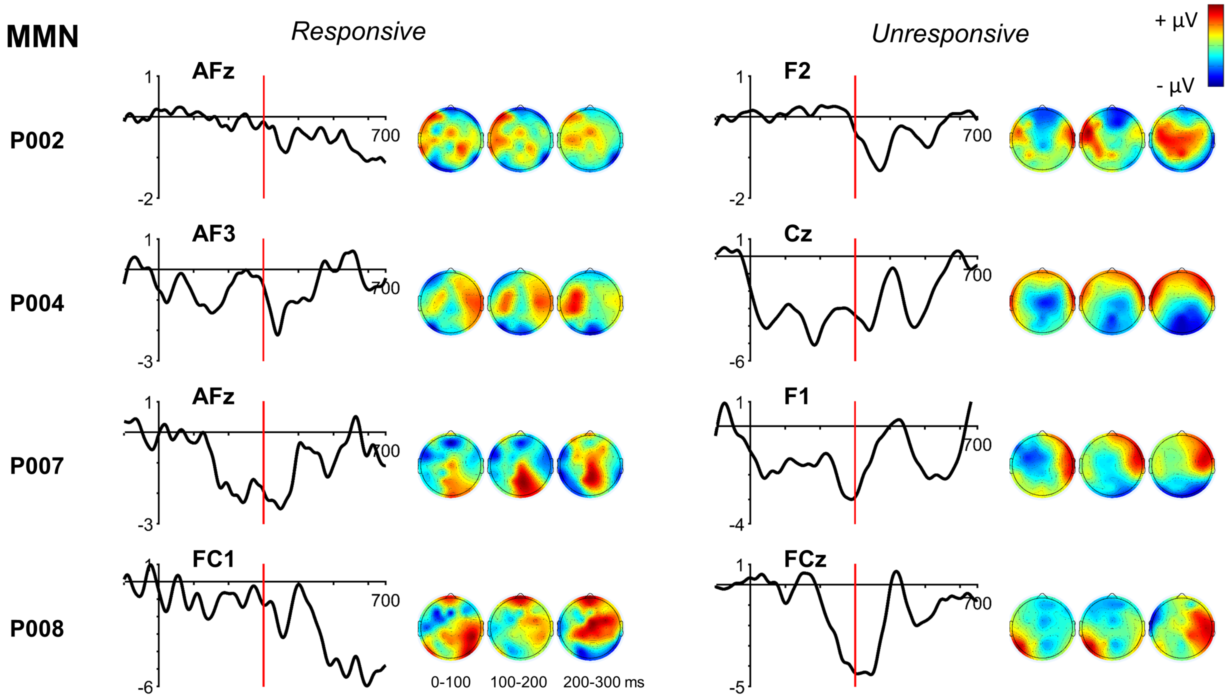

Supplementary Figure S4: Individual ERPs and Scalp Maps for the four patients who contributed data to both responsive and unresponsive sessions (MMN). Topographic data were averaged across each 100ms time interval. Scalp maps are scaled relative to their own minimum and maximum values. Only ERP difference waves from – 800ms to 700ms from the last tone of the run are shown. Difference waves were baseline corrected from -800ms to -600ms from the last tone of the run (i.e. -200 to 0ms from the first tone of the run) and filtered at 10Hz. Black bars represent the onset of the first tone of the run (-600ms), red bars represent the onset of the last tone of the run (0ms).

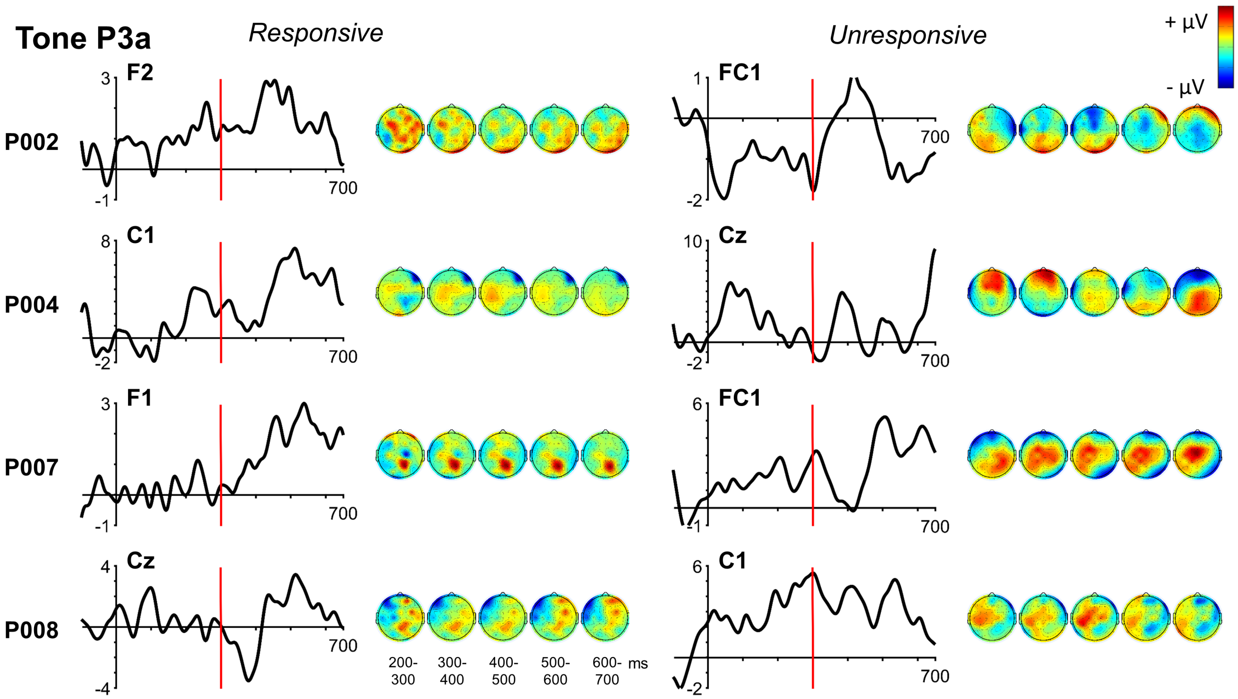

Supplementary Figure S5: Individual ERPs and Scalp Maps for the four patients who contributed data to both responsive and unresponsive sessions (Tone P3a). Topographic data were averaged across each 100ms time interval. Scalp maps are scaled relative to their own minimum and maximum values. Only ERP difference waves from – 800ms to 700ms from the last tone of the run are shown. Difference waves were baseline corrected from -800ms to -600ms from the last tone of the run (i.e. -200 to 0ms from the first tone of the run) and filtered at 10Hz. Black bars represent the onset of the first tone of the run (-600ms), red bars represent the onset of the last tone of the run (0ms).

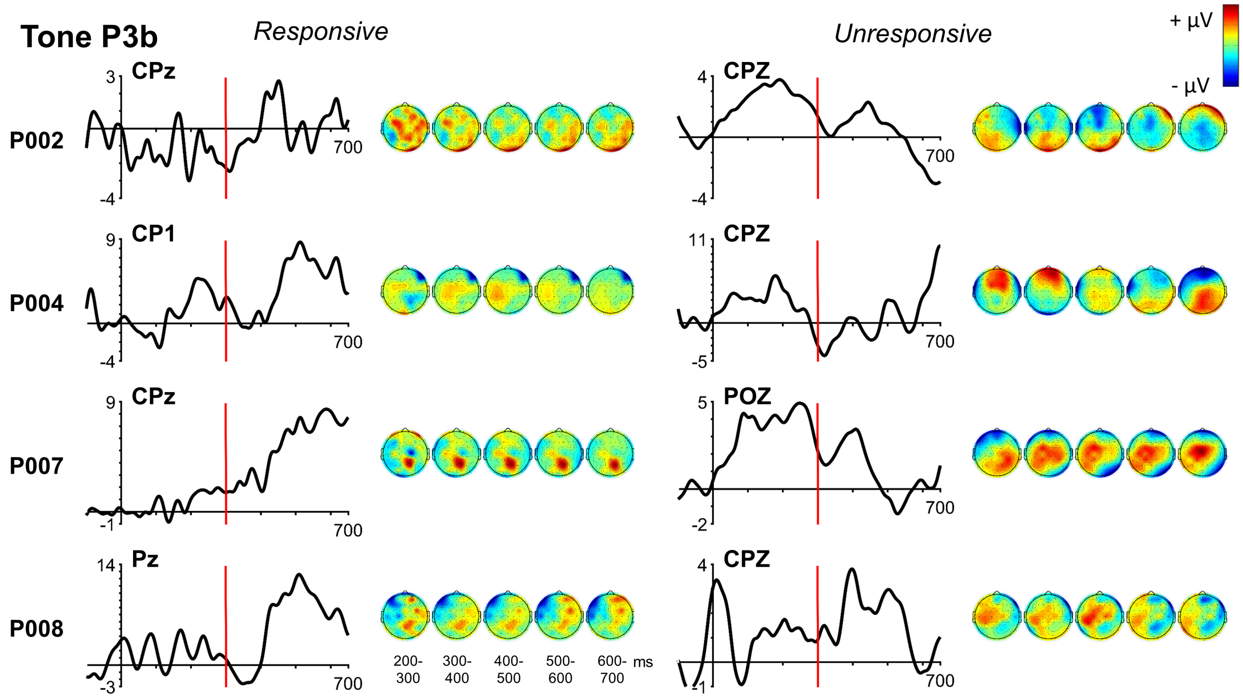

Supplementary Figure S6: Individual ERPs and Scalp Maps for the four patients who contributed data to both responsive and unresponsive sessions (Tone P3b). Topographic data were averaged across each 100ms time interval. Scalp maps are scaled relative to their own minimum and maximum values. Only ERP difference waves from – 800ms to 700ms from the last tone of the run are shown. Difference waves were baseline corrected from -800ms to -600ms from the last tone of the run (i.e. -200 to 0ms from the first tone of the run) and filtered at 10Hz. Black bars represent the onset of the first tone of the run (-600ms), red bars represent the onset of the last tone of the run (0ms).

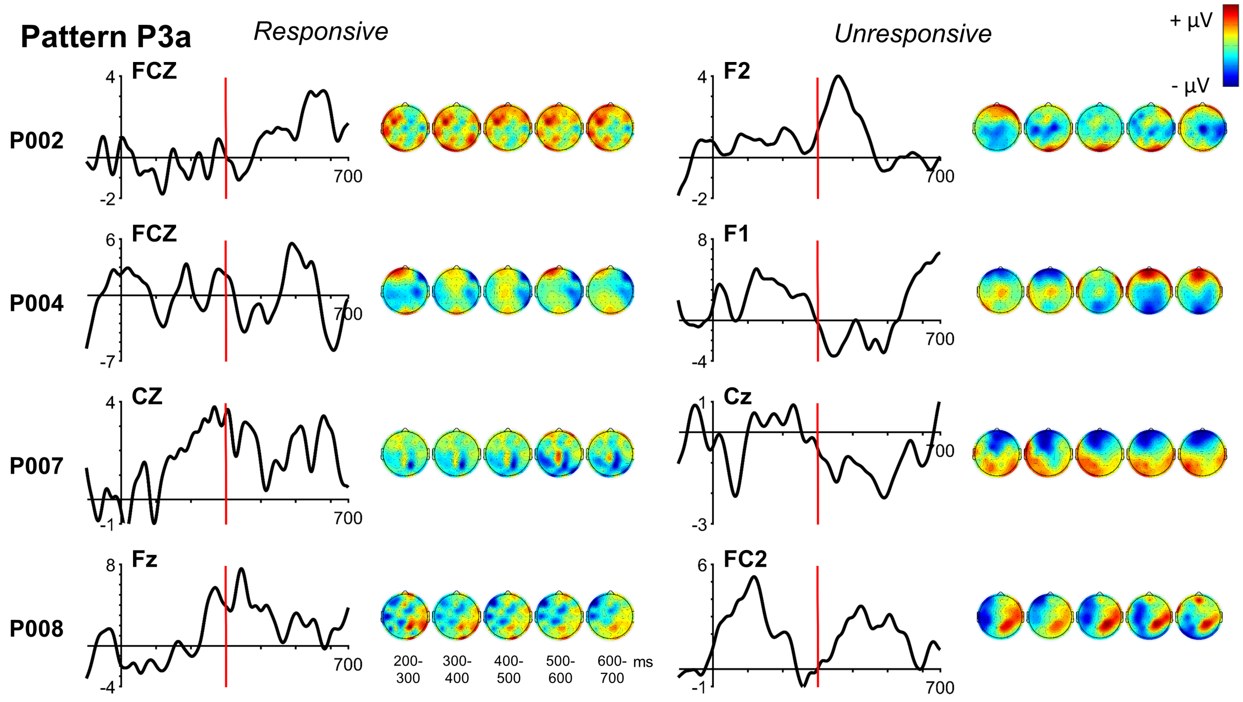

Supplementary Figure S7: Individual ERPs and Scalp Maps for the four patients who contributed data to both responsive and unresponsive sessions (Pattern P3a). Topographic data were averaged across each 100ms time interval. Scalp maps are scaled relative to their own minimum and maximum values. Only ERP difference waves from – 800ms to 700ms from the last tone of the run are shown. Difference waves were baseline corrected from -800ms to -600ms from the last tone of the run (i.e. -200 to 0ms from the first tone of the run) and filtered at 10Hz. Black bars represent the onset of the first tone of the run (-600ms), red bars represent the onset of the last tone of the run (0ms).

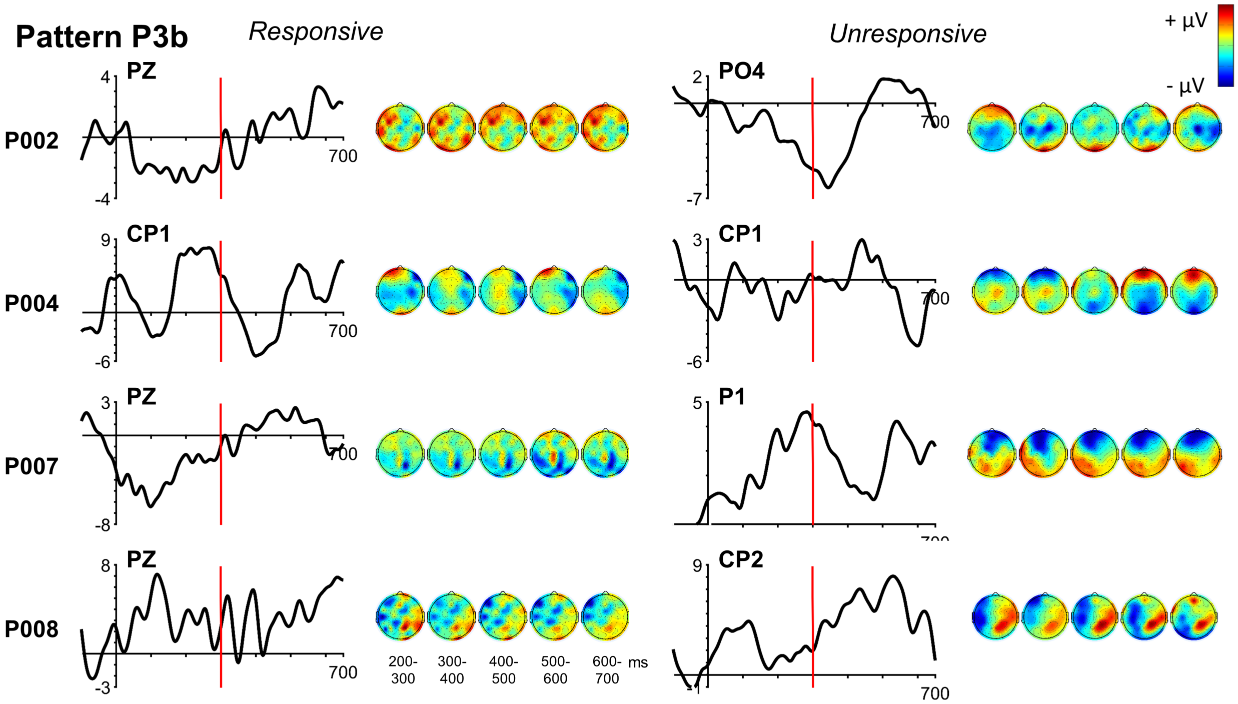

Supplementary Figure S8: Individual ERPs and Scalp Maps for the four patients who contributed data to both responsive and unresponsive sessions (Pattern P3b). Topographic data were averaged across each 100ms time interval. Scalp maps are scaled relative to their own minimum and maximum values. Only ERP difference waves from – 800ms to 700ms from the last tone of the run are shown. Difference waves were baseline corrected from -800ms to -600ms from the last tone of the run (i.e. -200 to 0ms from the first tone of the run) and filtered at 10Hz. Black bars represent the onset of the first tone of the run (-600ms), red bars represent the onset of the last tone of the run (0ms).
